# Burrow webs: Clawing the surface of interactions with burrows excavated by American badgers

**DOI:** 10.1002/ece3.7962

**Published:** 2021-07-30

**Authors:** Megan L. Andersen, Drew E. Bennett, Joseph D. Holbrook

**Affiliations:** ^1^ Haub School of Environment and Natural Resources University of Wyoming Laramie Wyoming USA; ^2^ Department of Zoology & Physiology University of Wyoming Laramie Wyoming USA

**Keywords:** American badgers, burrow web, ecological network, ecosystem engineers, species interactions, subterranean habitat, *Taxidea taxus*

## Abstract

Ecosystem engineers are organisms that influence their environment, which includes alterations leading to habitat provisioning for other species. Perhaps the most well‐examined guild of species provisioning habitat for other species is tree cavity excavators or woodpeckers (Picidae). Many studies have examined the suite of secondary cavity users that rely on woodpeckers, and how the ecological network of secondary users, collectively referred to as the *nest web*, changes across communities. Despite similar habitat provisioning processes, fewer studies have assessed the suite of species associated with burrowers providing access to subterranean habitat. Here, we begin to characterize the *burrow web* provisioned by American badgers (*Taxidea taxus*) and evaluate the diversity and frequency of species interactions we detected at abandoned badger burrows in Wyoming, USA. We deployed camera traps at 23 badger burrows and identified interactions with the burrow by birds, mammals, and reptiles. Overall, we discovered 31 other species utilizing badger burrows, consisting of 12 mammals, 18 birds, and 1 reptile. Mammals, other than American badgers themselves and other fossorial species such as ground squirrels (*Urocitellus* sp.), frequently using burrows included mice (*Peromyscus* sp.), long‐tailed weasel (*Mustela frenata*), pygmy rabbit (*Brachylagus idahoensis*), and desert cottontail (*Sylvilagus audubonii*). Of the 18 bird species detected, most accounted for <5% of overall detections, besides chipping sparrows (*Spizella passerina*) at 7.2%–11.5% of detections. The most common category of detection by bird species was foraging, contrary to mammals, which used the burrow frequently and were commonly observed entering and exiting the burrow. This work provides additional context on the ecological role of American badgers within their environment. More broadly, this work scratches the surface of many remaining questions to explore with the aim of advancing our understandings about *burrow webs* across the diversity of burrowing species and the communities in which they occur.

## Graphical abstract

Little work has assessed the suite of species associated with burrowing animals that provide access to subterranean habitat. Here, we characterize the burrow web provisioned by American badgers (*Taxidea taxus*), where we detected many birds and mammals interacting with abandoned burrows. Building on these initial observations will aid understandings about burrow webs across the diversity of burrowing species and the communities in which they occur.

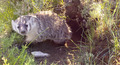

## 1 INTRODUCTION

Ecosystem engineers are organisms that influence their environment and affect resources for other organisms in the same habitat (Desbiez & Kluyber, 2013; Jones et al., 1994, 1997). These influences vary between species, depending on ecological roles and type of engineering. Autogenic engineers provide ecological resources via their physical self (e.g., a tree growing in a forest), whereas allogenic engineers change the physical state of an ecosystem through mechanical means (Jones et al., 1994). African elephants (*Loxodonta africana*) are an example of an allogenic engineer, shaping their ecosystems in various ways, including stripping a landscape of woody vegetation and promoting grasslands, enlarging water resources by further excavation, and dispersing seeds through consumption and defecation (Haynes, 2012). Similarly, gopher tortoises (*Gopherus polyphemus*) dig large yet shallow burrows, which are then further developed by other species including rodents and invertebrates. These secondary and tertiary burrow dwellers can rely heavily on the initial excavation provided by gopher tortoises to access subterranean habitat (Kinlaw & Grasmueck, 2012). Indeed, allogenic ecosystem engineers can fill important ecological roles, yet only a small set of species have been closely examined.

Perhaps the most well‐examined guild of allogenic engineers is tree cavity excavators or woodpeckers (Picidae). A number of studies have examined the suite of secondary cavity users that rely on cavity excavators and how the network of secondary users changes with body size (and thus cavity size) of cavity excavators (Cockle et al., 2011). The network of interactions between cavity excavators and secondary cavity users has been termed the *nest web* (Martin & Eadie, 1999). For instance, an assessment of nest webs in ponderosa pine (*Pinus ponderosa*) forests discovered that eight different species of cavity excavators of varying body sizes occurred within these forests, benefitting approximately 100 different species of cavity dwellers (Vierling et al., 2018). In a similar study from South America, authors discovered that forest composition surrounding cavities also affected how many species used tree cavities. For example, in a pewen (*Araucaria araucana*) forest, 26 birds and six species of mammals used cavities compared to 54 birds and five mammal species in a Parana pine (*Araucaria angustifolia*) forest (Cockle, Ibarra, et al., 2019). Finally, in a study from Canada within mixed conifer and deciduous forests, as well as aspen (*Populous tremuloides*) groves, over 32 secondary nesting species used cavities created by seven different species of cavity excavating birds (Martin et al., 2004). Collectively, this work has identified the essential role of multiple woodpecker species in engineering habitat for a suite of many other species within their ecological community. Despite the similarity in function to cavity excavators, comparatively fewer studies have examined the role of burrow excavation on the suite of co‐occuring species within differing communities (Davidson et al., 2012). Some notable exceptions include assessments of burrows excavated by prairie dogs (*Cynomys* sp.) and kangaroo rats (*Dipodomys* sp.; Davidson et al., 2008), bettongs (*Bettongia lesueur*; Read et al., 2008), aardvarks (*Orycteropus afer*; Whittington‐Jones et al., 2011), greater bilbies (*Macrotis lagotis*; Dawson et al., 2019), giant armadillos (*Priodontes maximus*; Blanco et al., 2020), and gopher tortoises (Murphy et al., 2021). Many other burrow excavators remain (e.g., Davidson et al., 2012), however, and additional work is required to expand the understanding of how burrowing animals influence ecological communities.

In North America, a prolific burrow excavator is the American badger (*Taxidea taxus*, hereafter badger). Badgers excavate burrows for foraging, resting, and denning (Bylo et al., 2014). Badgers prey upon ground squirrels (*Urocitellus* sp.), prairie dogs (*Cynomys* sp.), and other ground‐dwelling organisms (Eldridge, 2004; Grassel et al., 2015; Holbrook et al., 2016; Messick & Hornocker, 1981), primarily through the excavation of the prey species’ burrows. The denning burrows of badgers exhibit a diversity of subterranean structures; they are generally larger and more complex than foraging excavations. The most complex burrow systems excavated by badgers are those used for wintering, as well as the natal burrows in the spring (Symes et al., 2019). Burrows are excavated year‐round, but only a small proportion are occupied by badgers at any point in time, leaving many vacant burrows behind for potential use by other burrow dwellers (Eldridge, 2004). Estimates indicate badgers can generate up to 790 burrows/ha (Eldridge, 2004; Holbrook et al., 2016), which generally have 1–2 entrances (Symes et al., 2019) as opposed to other species like ground squirrels and prairie dogs where many entrances exist. Burrowing activity has biophysical consequences on soil properties such as texture, fertility, bulk density, and porosity (Eldridge, 2004; Eldridge & Whitford, 2009), as well as the distribution of water and other resources for vegetation (Kucheravy et al., 2021; Kurek et al., 2014). The magnitude of burrow excavation by badgers, as well as other species such as prairie dogs, also provides substantial subterranean habitat resources for the community of nonfossorial species. In areas where badgers and ground squirrels are the primary excavators, burrowing owls (*Speotyto cunicularia*) commonly use abandoned badger burrows for nesting and rearing of young; where prairie dogs are present, however, burrowing owls will also use prairie dog burrows for nesting (Desmond & Savidge, 1996). Despite the demonstrated importance of subterranean habitat to other, nonfossorial species, there have been few assessments evaluating the suite of species that secondarily use burrows excavated by the diversity of burrowers, including American badgers.

**FIGURE 1:**
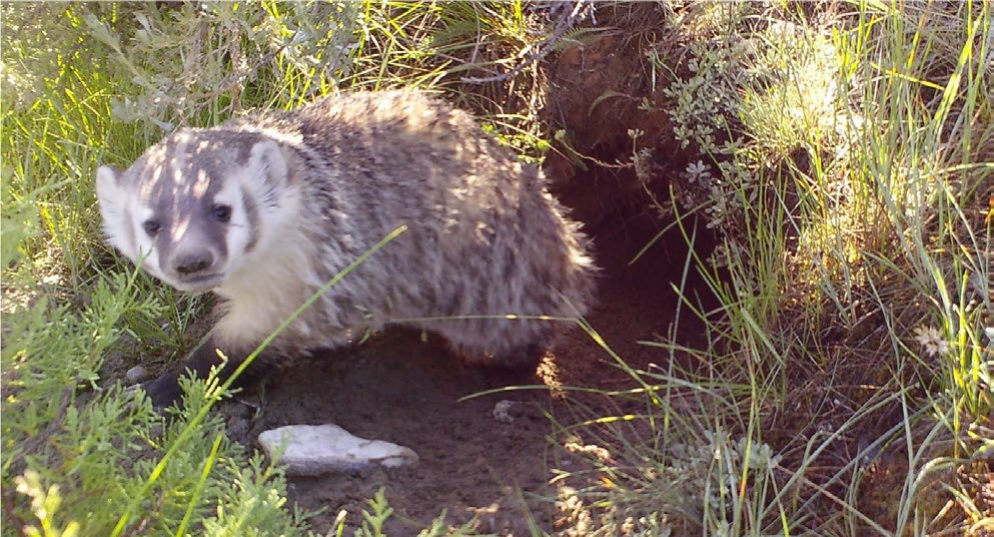
An American badger (*Taxidea taxus*) visits a burrow near Meeteetse, Wyoming, USA

Here, we began to explore the *burrow web* associated with American badgers (Figure 1). We were interested in documenting the number of species and frequency of interactions associated with burrows excavated by American badgers. Our main objectives were to (a) identify nonfossorial species that use abandoned badger burrows, (b) explore the differing functional uses of badger burrows by these species, and (c) determine which species used badger burrows most frequently. Within our study system (i.e., sagebrush‐steppe ecosystem), we generally expected mammalian species would be the dominant group using abandoned badger burrows, relative to other taxa such as reptiles or birds. However, this work was exploratory in nature, and thus, our expectations were mostly based on anecdotal evidence.

## 2 MATERIALS AND METHODS

Our study took place in high‐altitude (1,800–2,133 m asl) sagebrush‐steppe ecosystem of western Wyoming, USA, during the summer (June‐August) of 2019. One study location was around Pinedale (lat: 42.867, long: −109.861), Wyoming, while a second location was established near Meeteetse (lat: 44.158, long: −108.855), Wyoming. The two areas were about 220 km apart and were on private, working ranches with similar soil and climate characteristics (Natural Resource Conservation Service, 2019, 2020). We sampled both areas to capture some variation in plant and animal communities within the sagebrush‐steppe. In both study areas, we visually surveyed transects to detect evidence of badger activity, which was characterized by burrows with round or oval entrances of 16–30 cm in diameter and a mound of soil outside the entrance that was fan‐shaped (Eldridge, 2004; Holbrook et al., 2016). Both locations exhibited substantial badger activity as indexed by high burrow densities; additionally, we observed an individual badger foraging in each location. Our goal was to identify nonfossorial species that used abandoned (i.e., recently vacated) burrows excavated by badgers. We focused on abandoned burrows primarily because we expected the presence of badgers to have a negative effect on other species using the burrow. To classify abandoned burrows from recently excavated burrows (i.e., presumably occupied, or recently occupied), we assessed if the excavated mound had loose or crusted soil, cracks in the soil, or colonized vegetation (Eldridge, 2004; Holbrook et al., 2016). If we observed substantial crust or cracks in the soil (indicative of weathering), or colonized vegetation, we classified the burrow as abandoned (i.e., no evidence of relatively recent badger occupation). However, it is important to note that our classification of abandoned was based on evidence over a short temporal window (e.g., from the last rain storm altering the soil) immediately prior to our visual assessment. For our sampling, we considered any badger excavation, which could have included foraging, resting, natal, or wintering burrows.

**FIGURE 2:**
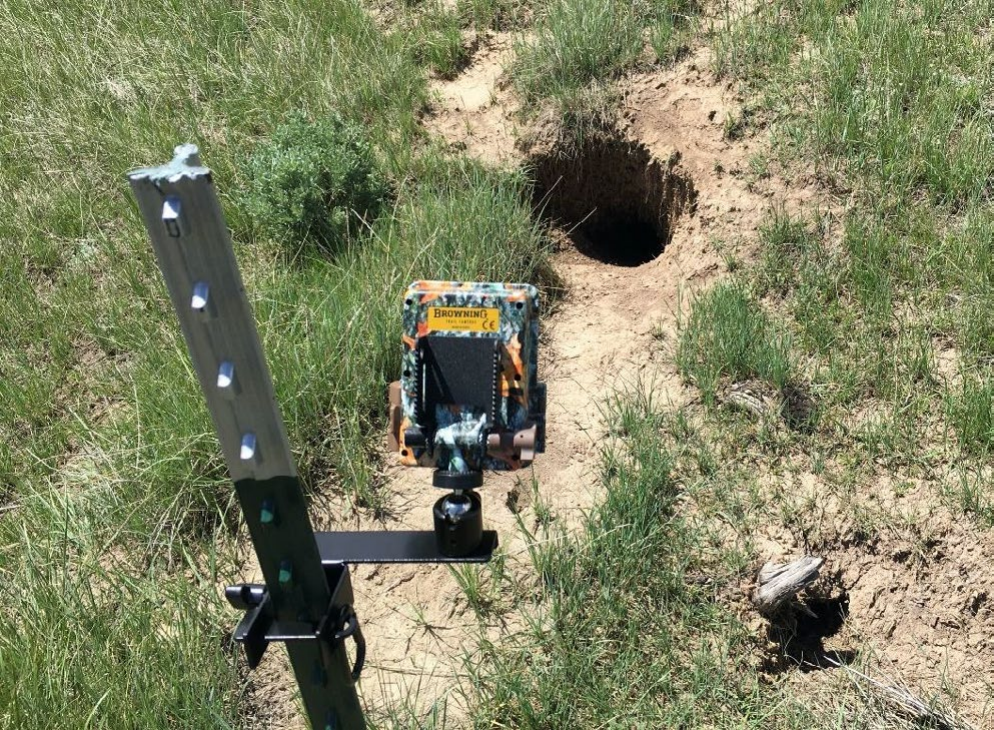
Example camera set‐up in front of an abandoned burrow excavated by an American badger (*Taxidea taxus*)

We systematically deployed a series of 23 camera traps (Browning, Strike Force HD Pro) at burrow entrances ≥10 m from one another. Ten cameras were set up in the Meeteetse location, while thirteen cameras were established in the Pinedale location. We mounted cameras to T‐posts (i.e., a metal stake 1.68 m tall) 0.60–0.90 m above the ground and ≈2 m in front of the burrow (Figure 2). We positioned cameras on T‐post mounts to include the entire entrance and mound (in front of the burrow) within the field of view. This positioning ensured we captured most vertebrates (e.g., mice were detectable) using the burrow entrance or mound. Detected invertebrates were not assessed because we were not confident in the camera's ability to capture all activity, even though we did detect many invertebrates. Given these detections, we assumed a detection probability at, or close to, 1 for vertebrates within the field of view of our cameras. Cameras were set to operate 24 hr a day, take a single image every time the sensor was triggered, and to continue taking images every 5 s with consistent triggering. We visited each camera approximately every 3 weeks to change SD cards and provide routine maintenance, including readjusting camera positions if they had been displaced. Most cameras operated continuously during our deployment, with a total of 1,076 trap nights and an average of 46.78 nights per camera (SD = 16 nights).

We processed images from each camera, and those containing vertebrates were saved and categorized as either mammals, birds, or reptiles and later identified to species (or genus if species was not possible). Each image of a vertebrate was then classified in one of six use categories (similar to Desbiez & Kluyber, 2013): in burrow (i.e., entering or exiting the burrow), inspect <5 s, inspect >5 s, moving, foraging, and bathing. Not only were we interested in the frequency of photographs (an index of time spent) across species, but also how the frequency of differing uses varied by taxonomic group and species. We classified images as in burrow if the organism was seen entering and/or exiting the burrow. Inspection was defined as short visits to the burrow without complete entrance, and further classified into less than or more than 5 s categories based on the number of images (the 5 s delay on the camera made this a simple calculation). Moving, foraging, and bathing all characterized activity outside the burrow for varying amounts of time. Once tabulated, we summarized our data in R (R Core Team, 2020) to examine abandoned burrow use by species.

We summarized frequency of detections for each species in two ways. First, we used the overall photographs taken as an index of time spent by a particular species exhibiting a particular use type (mentioned previously) at an abandoned badger burrow. Second, we summarized the frequency of discrete interaction events for each species at the burrows. We defined an interaction as a detection, or series of detections, where no other detection occurred 5 min prior to or after the detection(s). In other words, we reduced the frequency of total detections to discrete interaction episodes. We used both of these characterizations to construct a visual representation of the burrow web (similar to the nest web in Martin et al., 2004). We developed one visualization for the percent time spent and one for the percent of interactions. Ground squirrels and badgers were excluded from our visual representations because (a) they can excavate their own burrows (and we were primarily interested in those species that cannot), and (b) they were the most frequently observed. Omitting badgers and ground squirrels from our visuals allowed for closer examination of the other species using burrows, many of which were nonfossorial.

## 3 RESULTS

In total, we captured 33,119 images of vertebrates from the 23 burrows, and we identified 33,067 to genus or species (Table 1). We observed 31 species other than badgers at badger burrows, including 12 mammals, 18 birds, and 1 reptile (Table 1). We identified the lone reptile as a common garter snake (*Thamnophis sirtalis*). Of the 12 mammalian species, two species of ground squirrels (Wyoming ground squirrel and Unita ground squirrel, *Urocitellus ellegans* and *Urocitellus armatus*, respectively) accounted for 30,527 of the total images captured. The activity of both ground squirrel species was categorized primarily as in burrow. Badgers accounted for 679 of the total images and their activities included in burrow, inspection (<5 and >5 s), and moving. Of the 679 detections, 506 of them were from one location where a female badger with young occupied a burrow for a period of a month. Most other detections were characteristic of inspections, including brief use (e.g., photograph of a badger half in the burrow). Revisiting vacated burrows has been suggested as an effective foraging tactic for badgers, and our detection data indicated this might be true providing alternative evidence that other species (e.g., badger prey species) may commonly use presumed vacant burrows excavated by other species. For the remainder of our analyses, we exclude badgers and the two species of ground squirrels.

**TABLE 1:** Type and frequency of use at abandoned American badger (*Taxidea taxus*) burrows by all species observed

Common name	Scientific name	Group	Bathing	Foraging	Inspect (<5)	Inspect (>5)	In burrow	Moving	Total photographs	Interactions
Chipping Sparrow	*Spizella passerina*	Bird	11	55	55		13		134	91
Sparrow	*Passer* sp.	Bird		89				1	90	62
Brewers Blackbird	*Euphagus cyanocephalus*	Bird		67					67	48
Green‐tailed towhee	*Pipilo chlorurus*	Bird		24	36				60	39
Brewers Sparrow	*Spizella breweri*	Bird		50					50	35
Horned Lark	*Eremophila alpestris*	Bird		66					66	32
Magpie	*Pica pica*	Bird		22	7			1	30	16
Dark‐eyed Junco	*Junco hyemalis*	Bird		2	17				19	16
Sage Thrasher	*Oreoscoptes montanus*	Bird		25					25	14
American Robin	*Turdus migratorius*	Bird		16					16	12
Mountain Bluebird	*Sialia currucoides*	Bird		18					18	10
Vesper Sparrow	*Pooecetes gramineus*	Bird		12					12	8
Western Meadowlark	*Sturnella magna*	Bird		20					20	3
American Crow	*Corvus brachyrhynchos*	Bird			6				6	3
Savannah Sparrow	*Passerculus sandwichensis*	Bird		6					6	2
Common Poorwill	*Phalaenoptilus nuttallii*	Bird		4					4	2
Northern Flicker	*Colaptes auratus*	Bird						1	1	1
European Starling	*Sturnus vulgaris*	Bird		1					1	1
Peromyscus	*Peromyscus* sp.	Mammal		108			396		504	232
Long‐tailed weasel	*Mustela frenata*	Mammal				8	102	2	112	75
Least Chipmunk	*Tamias minimus*	Mammal		21	2		17	2	42	31
Jumping Mouse	*Zapus* sp.	Mammal		31					31	20
Pygmy Rabbit	*Brachylagus idahoenis*	Mammal					270		270	16
White‐tailed Jackrabbit	*Lepus townsendii*	Mammal			3		41		44	6
Microtus	*Microtus* sp.	Mammal	1	1			5		7	6
Desert Cottontail	*Sylvilagus audubonii*	Mammal				110			110	4
Peromyscus or Microtus	*Peromyscus*/*Microtus* sp.	Mammal			3				3	2
Pronghorn	*Antilocapra americana*	Mammal			2			93	95	1
Wyoming Ground Squirrel	*Urocitellus elegans*	Mammal			2		17,606		17,608	–
Uinta Ground Squirrel	*Urocitellus armatus*	Mammal					12,919		12,919	–
American Badger	*Taxidea taxus*	Mammal			93	9	576	1	679	–
Common Garter Snake	*Thamnophis sirtalis*	Reptile					18		18	1
Totals			12	638	200	127	31,744	101	33,067	789

Note

We used all photographs/detections to index time spent by species (i.e., total photographs) and type of use (i.e., bathing, foraging, inspect (<5), inspect (>5), in burrow, and moving). We reduced total detections to interaction events, which we defined as a detection, or series of detections, where no detections occurred 5 min prior to or after the detection(s); we did not assess interactions for ground squirrels (*Urocitellus* sp.) or American badgers (*Taxidea taxus*). Table is ordered based on taxonomic group (bird, mammal, reptile) and interactions.

**FIGURE 3:**
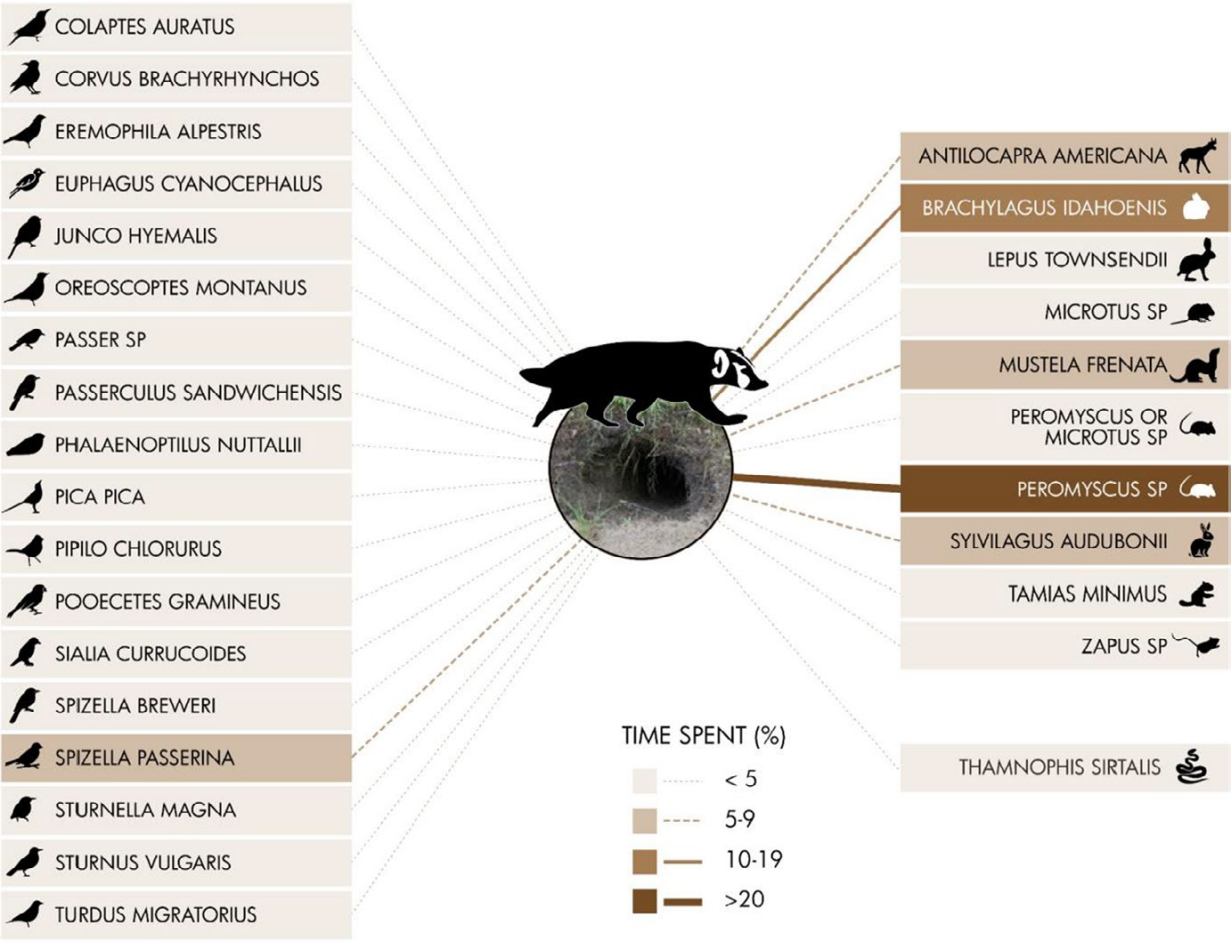
Frequency of use (i.e., index of time spent) at abandoned American badger (*Taxidea taxus*) burrows by all species (excluding badgers and ground squirrels). The single reptile species, a common garter snake (*Thamnophis sirtalis*), is listed on the lower right, below the mammal species. All bird species are listed on the left. This figure, displaying use by bird and mammal species, shows that mammals, specifically mice (*Peromyscus* sp.) and pygmy rabbits (*Brachylagus idahoenis*), made up the majority of time spent

We identified nine other mammal species interacting with abandoned badger burrows (Figure 3). Mice (*Peromyscus* sp. alone) spent the most time at the burrow, with 27.1% of detections. We observed pygmy rabbits (*Brachylagus idahoenis*) frequently at one camera location, making up 14.5% of time spent. Pygmy rabbits are semifossorial, but were of interest because of their conservation significance; for instance, they were petitioned for listing under the Endangered Species Act in 2008 (United States Fish & Wildlife Service, 2008). The following mammals each accounted for 5%–9% of overall time spent, pronghorn (*Antilocapra americana*) = 5.1%, desert cottontails (*Sylvilagus audubonii*) = 5.9%, and long‐tailed weasels (*Mustela frenata*) = 6%. The 5 other species accounted for less than 5% of detections, which included white‐tailed jackrabbits (*Lepus townsendii*) = 2.4%, least chipmunks (*Tamias minimus*) = 2.3%, and various species of mice (*Peromyscus* sp., and *Zapus* sp.) or voles (*Microtus* sp.). Of the mammal‐only detections, the majority of mammal activity was categorized as in burrow (50%), foraging (13%), use or foraging (18%), and inspection >5 s (9.7%). We expected inspection >5 s to be overrepresented as a consequence of the definition, yet this activity remained low compared to in burrow and foraging. Pronghorn generally moved past abandoned burrows (97.8% of pronghorn photographs were of them moving), in that the majority of images capturing pronghorn were of their legs walking through the field of view. The majority of mice or voles appeared to be foraging around the burrows, with less than 5 detections of bathing (either in soil or in water) and inspection (<5 s) combined. The remaining mammal species we detected generally entered/exited the burrow, inspected it for longer than 5 s, or foraged in excavated soil.

The 18 bird species we observed at badger burrows collectively accounted for 695 detections (Table 1). Chipping sparrows (*Spizella passerina*) were identified at burrows most often (7.2%; Figure 3). The remaining 17 bird species accounted for less than 5% of total time spent, which included Brewer's blackbirds (*Euphagus cyanocephalus*), horned larks (*Eremophila alpéstris*), American robin (*Turdus migratorious*), Brewer's sparrow (*Spizella breweri*), and western meadowlark (*Sturnella magna*) as a few examples (Table 1). We were unable to identify some birds to species, resulting in the categorization of these images as sparrows (*Passer* sp.), which accounted for 4.8% of detections. Of the bird‐only detections, birds were primarily foraging around the entrance of the burrows, totaling 477 images (76% of bird detections). Other activities included inspection for less than 5 s (19.4%), in burrow (2%), bathing (1.8%; either in soil or in water), and moving (0.5%).

**FIGURE 4:**
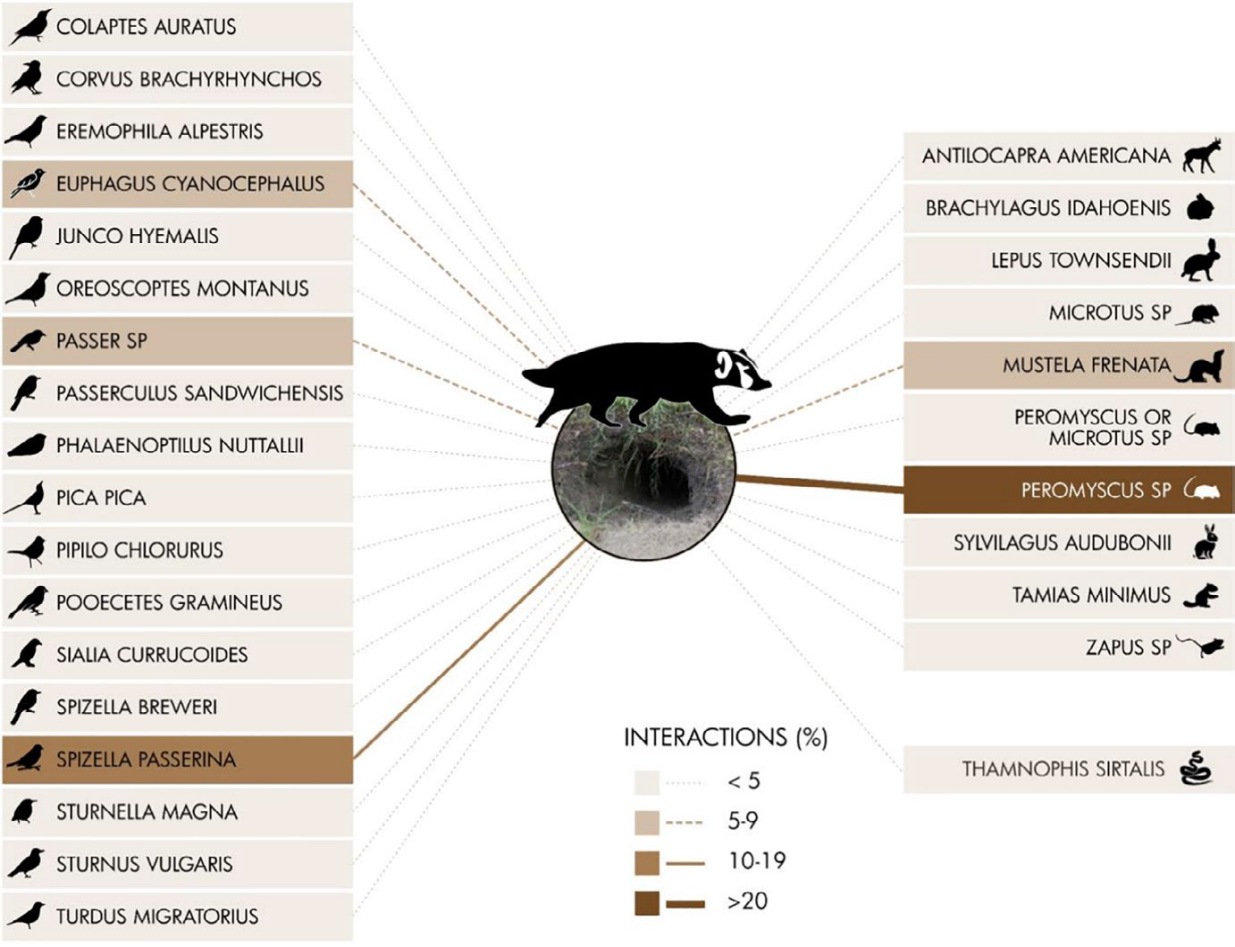
Frequency of interaction events at abandoned American badger (*Taxidea taxus*) burrows by all species (excluding badgers and ground squirrels). We defined an interaction as a detection, or series of detections, where no detections occurred 5 min prior to or after the detection(s). The single reptile species, a common garter snake (*Thamnophis sirtalis*), is listed on the lower right, below the mammal species. All bird species are listed on the left. This figure shows that both birds and mammals, specifically mice (*Peromyscus* sp.) and chipping sparrow (*Spizella passerina*), made up a substantial portion of interactions

Reducing our detection data into discrete interactions resulted in some changes to our burrow web (Table 1, Figure 4), despite the correlation coefficient between total detections and discrete interactions remaining high across species (*r* = .85, *df* = 27, *p* < .001). Similar to our assessment of time spent, we discovered that mice exhibited the highest number of interactions at burrows accounting for 29.4% of interactions (Figure 4). Interactions at badger burrows by chipping sparrow and long‐tailed weasel accounted for 11.5% and 9.5% of interactions, respectively (Figure 4), which was similar to our understanding of time spent. Sparrows (*Passer* sp.) and Brewer's blackbird were the next highest in terms of interactions with burrows (7.9% and 6.1%, respectively). The remaining mammal and bird species accounted for less than 5% of interactions (Figure 4), which included pronghorn, pygmy rabbits, and desert cottontail rabbits. These three species ranked relatively high in terms of time spent at badger burrows (Figure 3), but fell when assessing discrete interactions (Figure 4). The disparity between time spent and discrete interactions suggested that these species infrequently visited badger burrows, but when they visited they spent considerable time in front of our cameras.

## 4 DISCUSSION

Numerous studies have examined species interactions using an ecological network approach within food webs, host‐parasitoid webs, and mutualistic webs (Ings et al., 2009). Other interactions exist in ecological systems, and extending examinations to these interactions is essential to aid our understanding of ecological communities and their structure (Ings et al., 2009; Kéfi et al., 2012). Our work is among the first to begin clawing at the surface of potential interactions by nonfossorial species at burrows excavated by American badgers. However, much work remains to fully assess the suite of questions associated with the structure and importance of *burrow webs* in the broad sense.

**FIGURE 5:**
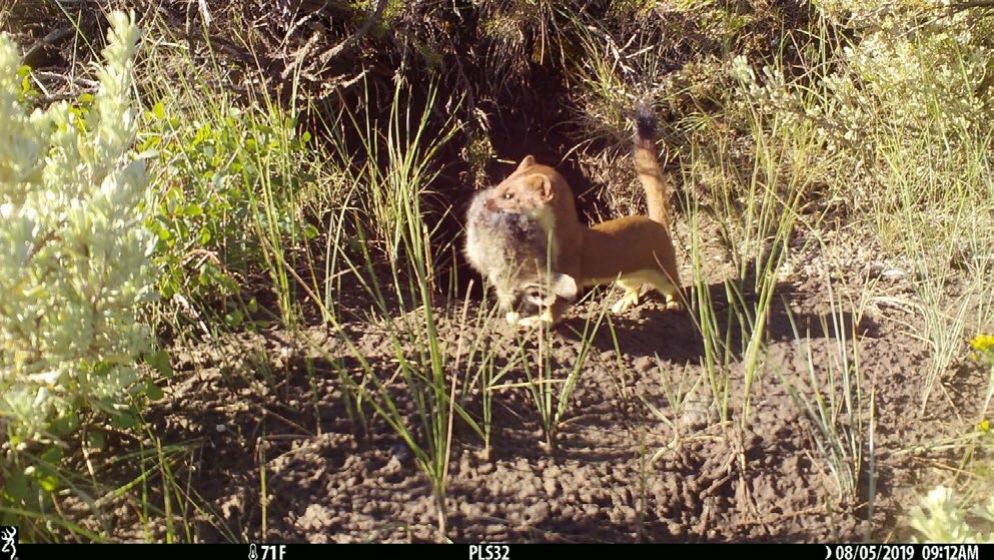
A long‐tailed weasel (*Mustela frenata*) carries a rabbit in its mouth after a presumed predation event near or within an abandoned burrow excavated by an American badger (*Taxidea taxus*)

We discovered a suite of species that were utilizing badger burrows for a variety of reasons, such as accessing subterranean habitat, foraging, inspection, and bathing (Table 1). Access to subterranean habitat may provide much‐needed thermal refugia for nonfossorial species in highly seasonal environments (Di Blanco et al., 2020; Symes et al., 2019; Whittington‐Jones et al., 2011), such as our study system (Milling et al., 2018; Symes et al., 2019). However, contrary to our initial thoughts, we observed many more bird species (compared to mammals) interacting with burrows. Based on the high foraging counts of birds, we hypothesize that the excavated mounds of soil outside abandoned burrows are a high quality or quantity environment for foraging. The exposed soil and microtopography sometimes created an ideal location for water collection for drinking as well as bathing. Soil piles also created an environment for dust bathing, which is an activity that some birds participate in to remove feather lipids, ectoparasites, or to regulate body temperature (Olsson & Keeling, 2005). Unexpected observations associated with abandoned badger burrows were predation events. For instance, we observed at least two instances of predation by long‐tailed weasels around abandoned badger burrows, one of which included a least chipmunk as the prey species while the other included a rabbit (Figure 5). We also observed American badgers revisiting and inspecting abandoned burrows frequently. This behavior may indicate an effective foraging tactic for badgers if prey species commonly use and occupy abandoned burrows (Grassel et al., 2015; Messick & Hornocker, 1981), which would further suggest that burrows are used by other species at a relatively high frequency. Burrow revisitation by badgers may also be a territory defense tactic, where badgers patrol burrows and scent mark; we observed one instance of this in our data collection. Collectively, we have provided a foundation to build upon to further characterize the extent and structure of the burrow web for American badgers, as well as generate synthetic comparisons with other fossorial species such as prairie dogs (Davidson et al., 2008), aardvarks (Whittington‐Jones et al., 2011), and giant armadillos (Blanco et al., 2020). Further, this work has provided new insights concerning the functional role of American badgers within their ecological community that extend beyond trophic interactions (Grassel et al., 2015) and biophysical impacts (Eldridge, 2004; Eldridge & Whitford, 2009).

A few studies have demonstrated the impact American badgers have on food webs (Grassel et al., 2015; Messick & Hornocker, 1981) as well as their surrounding environment through ecosystem engineering (Eldridge & Whitford, 2009). Badgers are adaptable carnivores that prey primarily on small mammals such as prairie dogs and ground squirrels, yet will consume reptiles, birds, and insects (Messick & Hornocker, 1981). In rangeland and desert environments, American badgers often assume the role of an apex carnivore and influence subordinate carnivores such as black‐footed ferrets (*Mustela nigripes*; Grassel et al., 2015). Studies have also examined the effects of biopedturbation by badgers on soil properties, determining that burrowing can have a lasting impact on soil structure, texture, fertility, bulk density, and porosity (Eldridge, 2004). These changes in soil characteristics can cascade to impact vegetation attributes such as species composition, as well as plant nutrient and water availability (Pagliai & Vignozzi, 2002), which may influence spatial behavior of herbivores such as pronghorn and rabbits.

Prior to our work, no study has explored the species that utilize abandoned badger burrows, despite similar questions being addressed for other fossorial mammals (e.g., Davidson et al., 2008; Dawson et al., 2019; Blanco et al., 2020; Read et al., 2008) along with the rich body of literature highlighting the cavity web (i.e., diversity and frequency of species secondarily using tree cavities) associated with woodpeckers (Cockle, Ibarra, et al., 2019; Cockle et al., 2011; Martin et al., 2004; Martin & Eadie, 1999). Our study has provided insight concerning the role of habitat provisioning by American badgers, which has substantial consequences on how we conceptualize badgers as ecosystem engineers. Despite our initial efforts to characterize species associated with abandoned badger burrows in the sagebrush‐steppe, there are known interactions absent from our assessment. For example, we did not observe nesting burrowing owls on our camera traps, but others have documented their use of abandoned badger burrows (Desmond & Savidge, 1996; Gleason & Johnson, 2016). According to Gleason and Johnson (2016), 75% of burrowing owls in a given population will utilize badger burrows for nesting sites, an essential feature for the reproductive success of owls. Along with burrowing owls, we did not identify any prairie dogs (i.e., white‐tailed prairie dogs, *Cynomys leucurus*) or black‐footed ferrets using these abandoned burrows, both of which are found in our study locations. More generally, we did not observe any lizard species using these burrows, nor did we sample the invertebrate community. Although our work is an important advancement with respect to habitat provisioning by American badgers, our insights are certainly an incomplete characterization. To fully capture the extent of habitat provisioning by badgers, additional studies in different habitats and communities, as well as longer duration assessments, are needed; this is particularly true given the extensive range of American badgers that spans numerous ecosystems across North America. Moreover, extending our questions to other fossorial animals that vary in body size and burrow architecture is required to fully characterize the extent, complexity, and structure of the burrow web.

When examining spatio‐temporal variation in burrow webs across differing communities, additional considerations include variation in soil types as well as the “life cycle” of the burrow. Analogs in the cavity web literature include variation in tree species and wood hardness, both of which influence cavity excavation by woodpeckers (Lorenz et al., 2015) and the life cycle of the cavity (Cockle, Trzcinski, et al., 2019; Edworthy et al., 2012). Previous work has indicated finer‐grained soils are more conducive to burrow excavation because they retain soil moisture better than coarse‐grained soils and are more structurally sound for complex burrow architecture due to the cohesive nature of clay and silt particles (Holbrook et al., 2016; Lohr et al., 2013). Initial work on burrow longevity has indicated that the lifetime of a burrow will likely increase in areas with reduced sand proportions (Goodman et al., 2018; Holmes et al., 2003). However, an exhaustive evaluation of burrow longevity across differing environments and a gradient in burrower body size has yet to be conducted. Variation in body size of the burrower might be a key factor when assessing the importance of different burrow uses to different species. For instance, if a prey species uses a burrow as escape habitat, it must be large enough for the prey yet small enough to limit access to the predator. Combining soil type, burrow longevity, and the suite of burrowers provides a transferable foundation to evaluate questions associated with the spatio‐temporal patterns of subterranean habitat across differing environments.

## 5 CONCLUSION

Burrowing animals are often ecosystem engineers that provide access to subterranean habitat for nonfossorial species, which is analogous to woodpeckers providing nesting and resting habitat within trees for many other species. Here, we provided the first assessment of species interacting with, and using, burrows excavated by American badgers. We documented approximately 31 species of mammals and birds interacting with badger burrows, which was somewhat counter to our initial thoughts. This suite of species interacted with badger burrows in a variety of ways including burrow entering/exiting, foraging, inspection, and bathing. Frequency of interactions changed by taxonomic group, with birds foraging and bathing most frequently, and mammals entering/exiting and foraging around the burrows. Collectively, this work provides additional context to the ecological role American badgers provide within their environment. Although informative, our work will only be complimented with additional studies examining the community of species associated with the gradient of burrowing animals across differing environments. A more refined and rigorous evaluation of burrow uses across nonfossorial species would also aid in characterizing the community‐level importance of access to subterranean habitat. Future work would benefit from assessments across differing communities that include a diversity of burrowers, increased spatial and temporal extents of evaluation, and the inclusion of variation in soil type and evaluations of burrow longevity. This would lead to a more holistic understanding of how burrowing animals influence the larger animal community and provide opportunities for synthetic comparisons between *cavity webs* and *burrow webs*.

## CONFLICT OF INTEREST

None declared.

## AUTHOR CONTRIBUTIONS

**Megan L. Andersen:** Conceptualization (equal); Data curation (equal); Formal analysis (equal); Investigation (equal); Methodology (equal); Visualization (equal); Writing‐original draft (equal); Writing‐review & editing (equal). **Drew E. Bennett:** Conceptualization (equal); Data curation (equal); Investigation (equal); Methodology (equal); Visualization (equal); Writing‐review & editing (equal). **Joseph D. Holbrook:** Conceptualization (lead); Data curation (equal); Formal analysis (equal); Investigation (equal); Methodology (equal); Visualization (equal); Writing‐original draft (equal); Writing‐review & editing (equal).

## Data Availability

Data are available on Dryad Digital Repository (https://doi.org/10.5061/dryad.z8w9ghxcq).
